# Chronic oligodendrocyte injury in central nervous system pathologies

**DOI:** 10.1038/s42003-022-04248-1

**Published:** 2022-11-19

**Authors:** Irene Molina-Gonzalez, Veronique E. Miron, Jack P. Antel

**Affiliations:** 1 United Kingdom Dementia Research Institute at The University of Edinburgh, Edinburgh, Scotland UK; 2 Centre for Discovery Brain Sciences, Chancellor’s Building, The University of Edinburgh, Edinburgh, Scotland UK; 3 Medical Research Council Centre for Reproductive Health, The Queen’s Medical Research Institute, The University of Edinburgh, Edinburgh, Scotland UK; 4 Barlo Multiple Sclerosis Centre and Keenan Research Centre for Biomedical Science, Toronto, Canada; 5 Department of Immunology, University of Toronto, Toronto, Canada; 6 Neuroimmunology Unit, Montreal Neurological Institute, McGill University, Montreal, QC Canada

**Keywords:** Neuroimmunology, Neurological disorders

## Abstract

Myelin, the membrane surrounding neuronal axons, is critical for central nervous system (CNS) function. Injury to myelin-forming oligodendrocytes (OL) in chronic neurological diseases (e.g. multiple sclerosis) ranges from sublethal to lethal, leading to OL dysfunction and myelin pathology, and consequent deleterious impacts on axonal health that drive clinical impairments. This is regulated by intrinsic factors such as heterogeneity and age, and extrinsic cellular and molecular interactions. Here, we discuss the responses of OLs to injury, and perspectives for therapeutic targeting. We put forward that targeting mature OL health in neurological disease is a promising therapeutic strategy to support CNS function.

## Abstract (web summary)

In this Perspective, Molina-Gonzalez and co-authors discuss the contribution of oligodendrocyte injury to neurological disease and the potential for their therapeutic targeting.

## Introduction

In the central nervous system (CNS), oligodendrocytes (OL) support neuronal health and function by extending myelinating processes and providing trophic and metabolic support to the underlying axon^1,2^. OLs are derived from the differentiation of oligodendrocyte progenitor cells (OPCs) throughout the lifespan^3–7^. However, most are generated in early development, within the first decade in humans and before 2 months of age in mice. Tracking of OL dynamics under homeostasis has indicated 100-fold more annual OL turnover in mice (>36%) vs humans (0.3%)^7^. What is consistent between species, however, is the highly dynamic turnover of myelin^4,5,7^. Injury to, and loss of, mature OLs and their processes are a prominent feature of myelin damage (demyelination) underpinning an array of clinical disorders across the lifespan.

The most common myelin disorder of the CNS is multiple sclerosis (MS), characterized by motor, sensory and cognitive impairments resulting from demyelinating lesions, considered to be initiated by transgression of immune constituents across blood-brain and cerebrospinal fluid (CSF) barriers. In active/demyelinating MS lesions, the numbers of mature OLs are comparable to the numbers in the NAWM, suggesting a relative preservation of OLs, in contrast to the significant loss in chronic lesions linked with disease progression^8,9^. Surviving OLs in acute lesions show “dying back” of processes with intact somas, suggesting sub-lethal injury that may precede their subsequent progressive loss^10^. This may be induced by systemic sources, such as fibrinogen^11^, that access the CNS via the blood-brain or CSF-brain barriers. In addition, there may be a contribution from local sources, including soluble products released by reactive microglia and astrocytes^12–15^ and the breakdown products of injured myelin^16–18^. Conversely, there could be lack of elements needed to maintain OL and myelin integrity (e.g., sources of energy, lipids)^19^. Discussed below in more detail are the potential mediators and mechanisms that underlie the above observations.

OL dysfunction/loss is now implicated in contributing to a wide array of neurological/psychiatric disorders. Pathogenesis of multiple system atrophy (MSA), characterized by a variable combination of parkinsonism, cerebellar impairment, and autonomic dysfunction, is ascribed to the accumulation of aggregated α-synuclein in OLs, which leads to severe neuronal loss, yet only a modest reduction in OLs^20,21^. Studies of the leukodystrophy Vanishing White Matter disease, in which mutations occur in subunits comprising the eukaryotic initiation factor 2B (eIF2B), demonstrate OL-directed cytotoxic function of astrocytes for reasons that are not completely understood^22^. Acquired pathology of OLs is also described in Parkinson’s Disease^23^, Alzheimer’s Disease^24^, and social isolation syndromes^25,26^, and dysfunctional OL-neuron interactions during development are described in Schizophrenia and Autism Spectrum Disorders^27–29^. There is evidence for varying susceptibility to injury based on the maturity of OL lineage cells. For instance, mature OLs are relatively preserved following perinatal white matter injury (e.g., periventricular leukomalacia, hypoxic-ischemic encephalopathy) in response to inflammatory and/or hypoxic-ischemic damage, as opposed to late-stage OL progenitors being highly susceptible to oxidative stress-induced apoptosis^30^. In addition, our analysis of MS tissues indicated a relatively greater loss of progenitors than mature OLs within active MS lesions^31^.

The OL/myelin deficits observed in the above neurological disorders highlight the critical need for therapeutics to preserve mature OLs, promote new oligodendrogenesis, and support the regeneration of myelin (‘remyelination’). In this Perspective, we cover the range of injury to OLs and intrinsic versus extrinsic influences on OLs, and reflect on how this knowledge may be harnessed for therapeutic development.

## Types of OL injury in MS

**Fig. 1:**
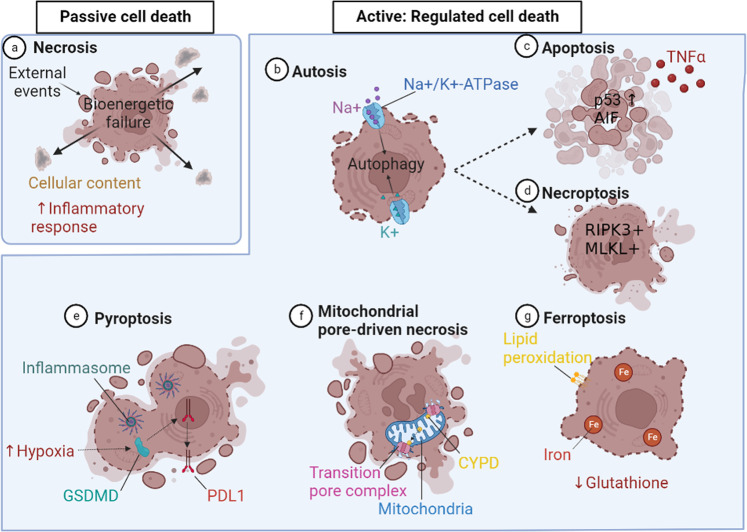
Types of cell death. Passive cell death; **a** Necrosis is a form of death where external events induce bioenergetic failure and plasma membrane breakdown, promoting the cellular contents to be released into the extracellular space, inducing an inflammatory response. Active: Regulated cell death; **b** Autosis is a form of excess autophagy mediated via Na^+^/K^+^-ATPase, which results in swelling and plasma membrane breakdown. **c** Apoptosis is regulated by the transcription factor p53 and is triggered by inflammatory mediators such as TNFα that activate the apoptosis-inducing factor mitochondria associated 1 (AIF). **d** Necroptosis is a form of controlled necrosis activated by death receptors like Toll-like and TNF receptor-1 (TNFR1) that act on RIPK1 and activate necroptosome forming elements, RIPK3 and MLKL. **e** Pyropotosis is a form of controlled cell death mediated by inflammasome activation, and triggered by hypoxia, activating gasderminD (GSDMD) leading to nuclear translocation of the programmed death ligand 1 (PDL1). **f** Mitochondrial permeability transition-driven necrosis is activated by metabolic stress which influences the opening of the permeability transition pore complex at the inner and outer mitochondrial membranes driven by the binding of cyclophilin D (CYPD). **g** Ferroptosis if a form of cell death that takes place under glutathione deficiency and induces an increase in cellular iron, altering the intracellular environment and causing lipid peroxidation. Created using BioRender.com.

Mechanisms underlying cell death have been categorized under two broad processes, namely passive or active (the latter also referred to as ‘programmed’ or ‘regulated cell death’) (1). As summarized by Galluzzi et al.^32^, the former commonly results from exposure to external events such as excessive temperatures, shear forces and/or pressures, and reflects mechanical disassembly of cellular constituents not involving molecular machinery. This cellular response is referred to as necrosis, and as stated by Edinger and Thompson^33^, is the end result of a bioenergetic catastrophe to a level incompatible with cell survival. Disruption of the plasma membrane induces cellular contents to escape to the extracellular space, evoking an inflammatory response. Apoptosis was the initial example of regulated cell death, involving a genetically programmed cell death that does not evoke a secondary inflammatory response. Now recognized are an array of regulated cell death processes, i.e., involving molecular mechanisms, many of which have now been demonstrated to contribute to OL death in experimental or in vitro models, and are being implicated in the MS disease process (Fig. 1). This category now also includes injury mechanisms that result in the features of cell necrosis described above.

### OL death in acute MS lesions

**Fig. 2:**
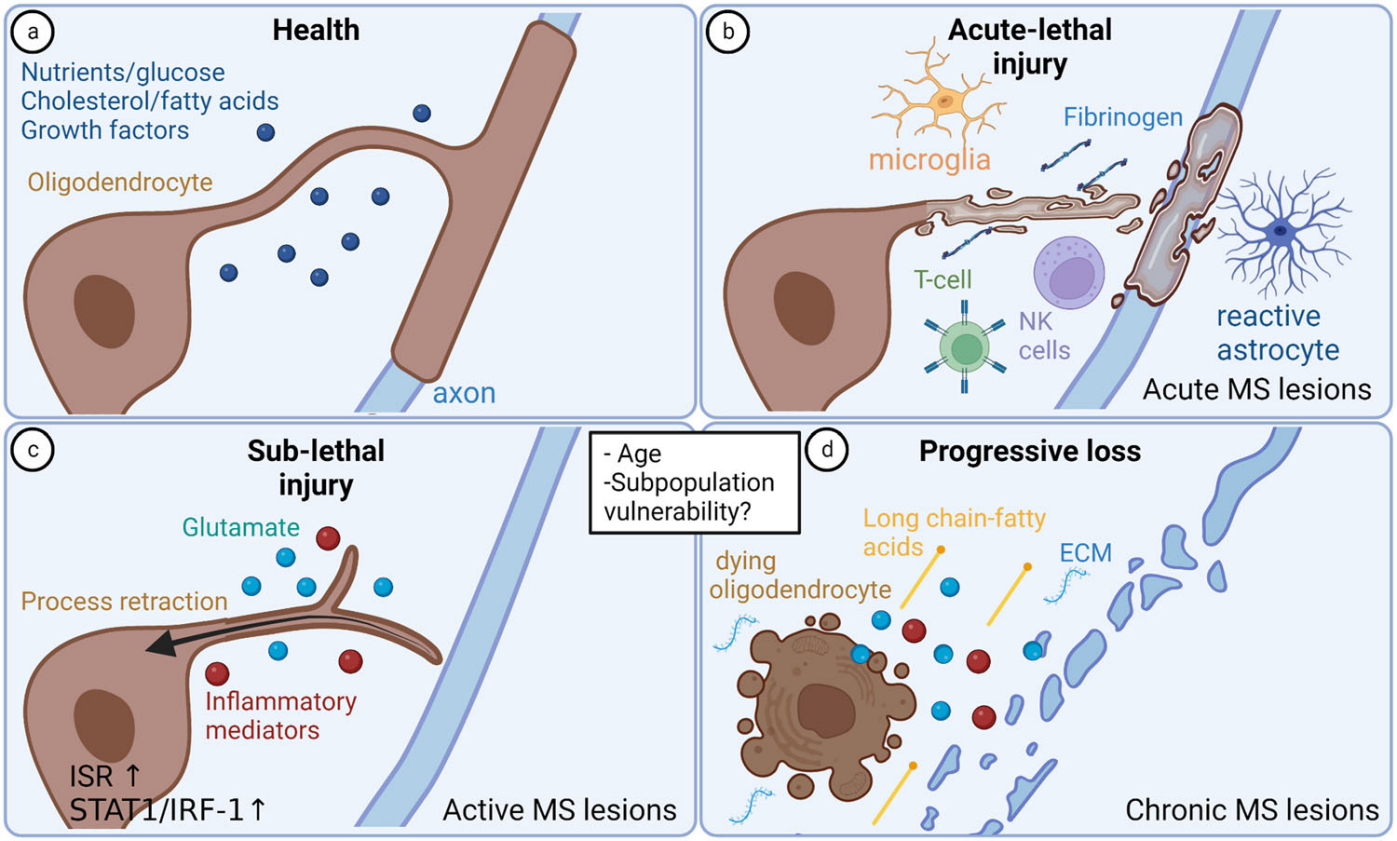
OL injury in Multiple Sclerosis. **a** Oligodendrocytes in healthy conditions rely on nutrients (e.g., glucose), lipids (e.g., cholesterol, fatty acids), and growth factors to support their survival and myelination. **b** Acute-lethal injury is observed in acute MS lesions driven by cytotoxic T and NK cells (contact dependent), reactive astrocytes, microglia and increased fibrinogen. **c** Sub-lethal injury to oligodendrocytes in active MS lesions involves process retraction with retention of cell bodies, which can be reversed by returning them to optimal conditions. Important contributors to sub-lethal injury include metabolic stress, glutamate, and inflammatory mediators. Susceptibility to such injury may be influenced by age, and oligodendrocyte subpopulations may be specifically vulnerable. **d** Combination of insults and age can convert sublethal responses to progressive loss. Ongoing loss of oligodendrocytes is regulated via cell death mechanisms during disease progression, changes to the extracellular milieu such as modifications to the extracellular matrix (ECM) and presence of cytotoxic long chain fatty acids. Created using BioRender.com.

Barnett and Prineas observed dying OLs in a small subset of brain stem lesions which showed morphological features typically associated with apoptotic cell death; however, only 2% of the apoptotic OLs were stated to be immuno-reactive for activated caspase-3^34^. The authors did put forward however, the challenges of detecting caspase-3 expression in OL in post-mortem tissue due to the potential rapid disappearance of dying cells, or tissue preservation artefacts. This OL pathology was stated to resemble the type III lesions described by Lucchinetti et al.^9,35^. A concept suggested by Casaccia and Bodekke is that OL death in MS lesions could reflect a continuum of injury mechanisms dependent upon the specific insults and its severity^36^. We have shown that multiple types of cytotoxic immune cells found in acute lesions, including γδ T cells, activated NK cells^37^, CD56 + NKG2C + CD4 + T cells^38^, and CD4 + Th17 cells^39^ can mediate non-MHC restricted cell-cell contact lethal injury of human adult brain-derived mature OLs in vitro (Fig. 2). In addition, Jamann et al. showed that for CD4 + Th17 cells, OL cytotoxicity was perforin/granzyme-dependent^40^.

### Sub-lethal OL injury

Rodriguez et al.^10^ identified dying back of OL processes with preserved cell bodies in acute MS lesion biopsies, as had first been described in early phases of demyelination in the cuprizone mouse model by Ludwin et al.^41^. Such retraction would impair participation in any recovery response. We reproduced this potentially reversible response by human adult mature OLs in both dissociated cell culture and microfibre ensheathment assays, using an array of insults implicated in MS pathogenesis including the pro-inflammatory molecules interferon (IFN)-γ and tumor necrosis factor (TNF)-α, the excitatory neutrotransmitter glutamate, and metabolic stress conditions involving nutrient deprivation and low glucose^19,42^. RNA sequencing revealed that process retraction can occur via a number of distinct mechanisms, reflecting the mediators of injury (Fig. 2). First, IFNγ or TNF-α induced the STAT1/IRF-1 signaling pathway; this pathway has been shown to impair process outgrowth in neurons and regulate glycolysis - the major source of of ATP in mature OLs which contributes to protein and lipid production needed for myelin biosynthesis. Some studies have also proposed that TNFα modulates the Rho GTPase pathways regulating the cytoskeleton through the activation of the autophagy pathway, providing a link to a stress response^42–44^. Second, glutamate-induced few changes in gene expression, implicating a direct effect on OL processes. Third, metabolic insult-induced OL process retraction was associated with induction of the integrated stress response (ISR)^42^, which can either be protective or contribute to cell injury, depending on its level and duration of activation. Addition of the ISR agonist Sephin1 to cultured human OL—maintained under control conditions or exposed to ongoing metabolic stress—reduced process outgrowth^42^, suggesting that the ISR regulates the protein synthesis machinery required for OL process outgrowth and maintenance. Consistent with this postulate, inhibiting the ISR after stress induction enhanced OL process outgrowth^42^. Expression of ElF2α, a major regulator of the ISR pathway, is increased in oligodendrocytes in active MS lesions compared to levels in normal appearing white matter or control tissue^42^.

Further to be considered are the effects of combined insults as a link between sublethal injury and subsequent OL cell death. As an example, we have previously shown that adenovirus-mediated induction in human adult OLs of low levels of p53, a transcription factor that regulates apoptosis and can be detected in OLs in actively demyelinating MS lesions, renders these cells susceptible to subsequent FasL- and TRAIL-medicated killing^45^. P53 can be induced by multiple stress stimuli, including inflammatory cytokines/inflammation and ischemia/hypoxia. As observed in OPCs in MS lesions^46^, it is tempting to speculate that cellular senescence may play a role in regulating OL responses, as senescent cells are resistant to cell death; this postulate remains to be investigated.

### OL loss in progressive MS

In contrast to acute lesions, there is consensus of significant oligodendroglial cell loss in the center of chronic active lesions as indicated by neuropathological observations of post-mortem tissue^8^ and single nuclei sequencing^47^. However, although a number of studies have tried to identify the pathways involved, the precise mechanisms of OL death remain to be established and are being investigated in ongoing studies using experimental in vivo and in vitro models. An increasing number of regulated cell death mechanisms are being defined by their distinct molecular signatures, and linked to specific inducing signals or conditions (Fig. 2). These pathways can be triggered by specific receptor engagement, such as by immune/infectious constituents and/or by metabolic insults, all of which are implicated in the MS disease process. For instance, apoptosis can be triggered by inflammatory signals or metabolic stress. However, Prineas and Barnett observed in two secondary-progressive MS cases that surviving OLs present amongst the microglia and macrophages at the lesion edge rarely showed apoptotic nuclei^48^.

Necroptosis is a form of necrosis that depends on a membrane pore-forming complex termed the necroptosome. Necroptosis is usually triggered via Toll-like receptors or death receptors that include TNF receptor-1 (TNFR1)^49^ acting via RIPK1 to sequentially activate RIPK3 and MLKL. Ofengeim et al. (2015) implicated activation of the necroptosis pathway as contributing to progressive MS, finding that ~50% genes upregulated in chronic active lesions are regulators of this process^50^. Necroptosome markers RIPK1 and MLKL were expressed in OLs and microglia, in MS lesions, the latter consistent with our findings of RIPK3+ and MLKL + microglia in remyelinating MS lesions^51^. *Ripk3*^−/−^ mice had increased resistance to cuprizone-induced demyelination, and OLs derived from these mice were less susceptible to TNF-α-mediated necroptosis^50^. However, Zhang et al. found that cuprizone-induced injury reflected an MLKL-dependent, but RIPK3-independent, mechanism of injury acting via myeloid cells^52^. Zelic et al. then showed that RIPK1 activation in microglia and astrocytes induces a detrimental neuroinflammatory program that contributes to the neurodegenerative environment in progressive MS^53^. Of note, we could not inhibit cell death in human mature OLs exposed to metabolic stress with necroptosis inhibitors^19^. Jurewicz et al. found that TNF treatment of human OLs in culture induces apoptosis-inducing factor mitochondria associated 1 (AIF)-mediated cell death, also known as parthanatosis, involving hyperactivation of DNA damage response machinery^54^. TNFα was also shown to induce human OL progenitor cells to undergo death by pyroptosis, a pro-inflammatory programmed cell death mediated by inflammasome complex activation^55^. OLs in MS lesions were shown to express a pyroptosis mediator, gasderminD (GSDMD), and inflammasome inhibition reduces injury to the OL lineage in EAE^55^. Hypoxia, a feature of a subtype of MS lesions^35^, can induce GSDMD expression through phosphorylation of STAT3, which promotes nuclear translocation of programmed death ligand 1 (PDL1)^56^.

Both histological and imaging analyses implicate metabolic stress as a contributor to lesion evolution in MS^57–64^, with reduced focal cerebral blood flow associated with chronic hypoxia, lesion formation and axonal degeneration, and evidence of axonal metabolic dysfunction. Autophagy is a process of degradation of cellular components by the lysosomal pathway as an adaptive response to stress, to mediate cytoprotective responses and enhance energy production. Blocking autophagy of metabolically stressed OLs in vitro by using chloroquine results in increased cell death^65^. This could potentially reflect reduced energy production and/or failure of the auto-phagosomal/lysosomal pathway to clear tissue injury-mediating products. Excess autophagy activation itself can contribute to cell death mediated via Na^+^/K^+^-ATPase, resulting in cell swelling and plasma membrane rupture (a process known as autosis)^66^ and by activating other selected cell death pathways, such as apoptosis^67^ and necroptosis^68^. Metabolic stress can also trigger mitochondrial permeability transition-driven necrosis which involves the opening of a supramolecular complex (‘permeability transition pore complex’) at the junctions between inner and outer mitochondrial membranes; OLs in MS lesions express cyclophilin D (CYPD), a driver of mitochondrial pore-driven necrosis; deletion protects against neurodegeneration in demyelinating experimental models of MS^69^.

Other types of cell death may be critical in regulating OL death but have not yet been demonstrably shown to do so in human demyelinating disease. Experimental OL death and demyelination induced by the copper chelator cuprizone involves ferroptosis^70^—an iron-dependent form of cell death induced by perturbations of the intracellular environment, leading to lipid peroxidation under conditions of glutathione deficiency. This likely reflects an impact on OL metabolism, as copper-containing iron-oxidizing enzymes (ferroxidases) are involved in mitigating oxidative stress. Indeed, OL loss in the cuprizone model can be rescued by treatment with a lipid radical scavenging molecule, ferrostatin 1^70^. Overall, the mechanisms by which human OLs undergo cell death are still being resolved, with the potential for new regulated cell death mechanisms to be uncovered and the development of novel therapeutic interventions. Whether these distinct forms of cell death related to distinct pathologies, CNS regions, age, or subpopulations of OLs remains to be defined.

## Intrinsic regulation of oligodendrocyte responses

**Fig. 3:**
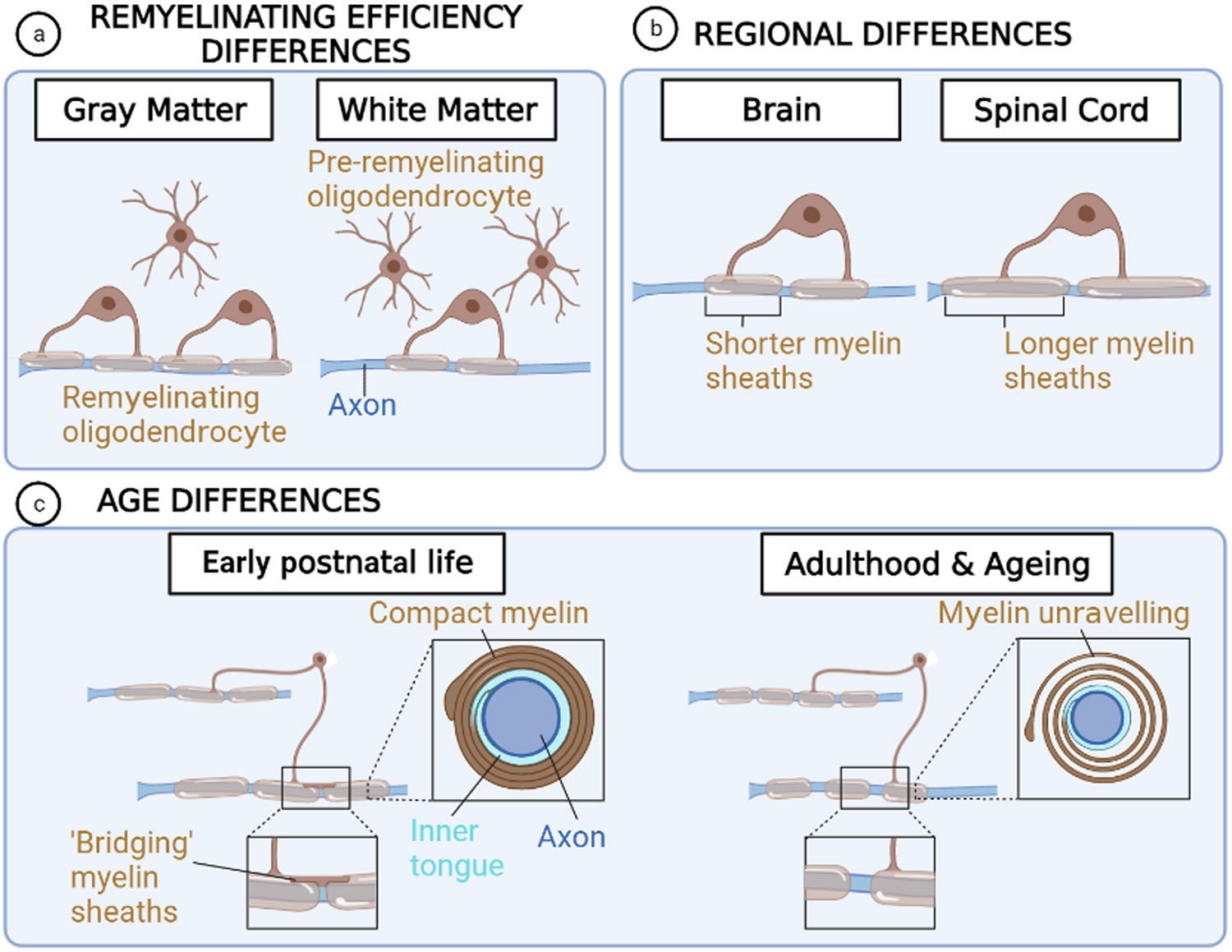
Oligodendrocyte Heterogeneity. **a** Remyelinating efficiency differences are observed between the gray matter and the white matter, oligodendrocytes in the former remyelinate more efficient than the latter, where oligodendrocytes have a higher capacity to differentiate. **b** Oligodendrocytes show regional differences, in the brain forming shorter myelin sheaths than in the spinal cord. **c** Oligodendrocyte differences exist during the lifespan, when during early postnatal life oligodendrocytes form compact myelin and longer myelin sheaths, and can create bridges forming sequential myelin sheaths known as ‘bridging myelin sheaths’. In adulthood and ageing, myelin sheaths are shorter and uncompact, leading to myelin unravelling and loss of the bridging myelin sheaths. Created using BioRender.com.

Multiple variables may influence the intrinsic properties of oligodendrocytes which in turn contributes to their responses to injury, including heterogeneity and age, and OL differentiation state (Fig. 3).

### Heterogeneity

OLs show intrinsic heterogeneity as has recently been demonstrated at the transcriptional level using single cell/nuclei RNA sequencing, with 6–9 subpopulations of OLs identified in mouse and human brain under healthy conditions and in the context of demyelination (EAE and MS)^71,72^. A significant amount of overlap between human and mouse clusters has been observed. Of interest, mature OL clusters expressing genes suggestive of immune function have been identified, for example supporting an MHC-class II-mediated response^71^. In this regard, a recent study revealed a human-specific OL subpopulation enriched in the spinal cord with predicted immune function^73^. Examples of regional differences in OL function include observations that spinal cord OLs form longer myelin sheaths on microfibres than their cortical counterparts, recapitulating in vivo myelin properties^74^. OLs show faster differentiation yet slower remyelination in white matter vs gray matter in mouse models and MS lesions, and reduced remyelination in spinal cord vs deep white matter^75–80^. However, white matter OL lineage cells are more intrinsically regulated vs those in the gray matter, as transplantations into the adult mouse cerebral cortex revealed that white matter-derived cells differentiated into mature OLs in both niches with equal efficiency, whereas gray matter-derived cells did not^75^. Moreover, a distinct OL population (‘peri-neuronal OL’) has been identified in gray matter regions^81^.

In the context of human neurodegenerative diseases such as MS and Alzheimer’s disease (AD), OLs undergo a shift in gene expression and subpopulations compared to healthy controls^71,82,83^. Single RNA sequencing of the most extensive MS cohort to date revealed differences in OL heterogeneity in white matter vs gray matter, and vulnerability of a specific OL subpopulation^84^. OL subpopulation profiles allowed for the stratification of MS samples into 3 types: (1) having OL subpopulations largely similar to that observed in normal appearing white matter, (2) showing an increase in an OL subpopulation with a stressed profile, and (3) indicating a block in OL differentiation^84^. Of note, pathway analysis of OLs in MS gray and white matter lesions indicated engagement of pathways which include those related to injury responses (e.g., p53 and metabolism)^84,85^.

### Age

Myelination is tightly regulated over the lifespan, with onset in humans in the third trimester and peaking in the first decade, and continuing on in adulthood to support learning and memory processes^86^. ^14^C -dating studies in humans indicate that OLs are long-lived cells, yet there is dynamic turnover of their myelin membranes^7^.

Myelin undergoes structural changes with age. In mice, OLs born later in life elaborate increased numbers of myelin internodes, yet these are shorter than those generated in early postnatal life^3^. We have shown that myelination capacity of human OL lineage cells decreases postnatally through to adulthood^87^. With adulthood and ageing, there is a reduced amount of new myelin formed in the context of a learning task or following demyelination^88,89^, in association with impaired oligodendrogenesis. In addition, myelin structure becomes dysregulated particularly in the white matter, with myelin thickening, outfoldings, unravelling, enlargement of inner tongues^90–92^. Degeneration of myelin sheaths occurs with aging^5^, with particular vulnerability of newly discovered ‘bridging’ myelin sheaths, by which OLs generate sheaths via an extension of the outer tongue of an existing sheath^93^. Age is associated with poor efficiency of remyelination in animal models, and is a predominant risk factor for MS progression^94^.

The molecular underpinnings of OL and myelin changes with ageing are still being elucidated. These findings may reflect in part the epigenetic changes in the OL lineage with ageing, which contribute to poor remyelination^95^. Transcriptional profiling of rodent OLs with ageing suggests increased engagement of pathways related to antigen presentation, phagocytosis, and interferon signaling^96,97^. Our own transcriptomic data indicated that pediatric human OLs (<5 years of age), while expressing mature lineage markers, still express developmental markers suggesting they are less mature than their adult counterparts^98^. The pediatric OLs showed increased expression of immune regulatory genes, as observed in comparative studies with mature OLs^99^. Our study also showed increased expression of metabolism-related genes in human adult vs pediatric OLs^98^, perhaps reflecting the extended state of myelin production in the former.

With regards to metabolism, under optimal conditions, adult rat OLs preferentially use glycolysis whereas in development, OPCs and newly differentiated OLs mainly utilize oxidative phosphorylation to produce Adenosine 5′-triphosphate (ATP). We observed that human mature OLs also preferentially use glycolysis but overall have a significantly lower rate of metabolic activity compared to their rat counterparts^100^. Neumann et al compared metabolic properties of OPCs in culture derived from young versus aged adult rats, and found lower ATP levels and cellular respiration in the latter, likely reflecting mitochondrial dysfunction^101^. Our in vitro studies indicated that mature human OLs derived from young-middle aged adult brain undergo non-apoptotic death in response to metabolic stress culture conditions in vitro^19^; conversely, OLs and progenitors from pediatric and fetal sources show a measurable apoptotic response, with differences ascribed to relative expression of effector versus protective members of the BCL-2 family^65^.

## Extrinsic regulation of oligodendrocyte responses

OL responses are regulated by extrinsic influences throughout the lifespan, including in response to CNS injury. This includes being dependent on factors in the microenvironment and cell-cell interactions for their health and function (Figs. 2, 3).

### Lipid regulation of OL responses

As OLs extend up to 60 myelinated processes and can generate up to 50 × 10^3^ µm^3^ of membrane per day, myelination and remyelination are highly demanding processes. Up to 80% of myelin membrane is formed of lipids as main structural components, and the integration of cholesterol and fatty acids into myelin membranes is a critical aspect of myelin growth during development and remyelination^102–104^. For instance, cholesterol in oligodendrocytes is an essential building block for efficient myelin growth^102^. However, OLs do not solely rely on their own production of lipids for myelination. Conditional deletion of genes in OLs critical for lipid synthesis leads to reduced OL number and impaired myelination, yet these eventually normalize, indicating compensation through uptake from neighboring cells^102,105^. Indeed, astrocyte conditional knockouts which impair their lipid synthesis in development result in hypomyelination, and myelin defects only persist when both OL and astrocyte lipid synthesis are inhibited^105^. However, dietary lipid supplementation improves neurological deficits caused by the lack of lipid generation by astrocytes^106^.

Furthermore, both cholesterol and fatty acids can act as signaling units and energy sources to regulate the survival of oligodendrocyte lineage cells in development and during remyelination (Fig. 2)^104,107^. They form membrane lipid rafts which house pro-survival receptors such as integrin receptors^108^. Cholesterol intermediates such as 8,9-unsaturated sterols and desmosterol have recently been shown to regulate OPC differentiation during myelination and early remyelination^109–113^. Microglia provide such intermediates to OPCs during remyelination^110^, and this can occur through transfer of lipid-containing extracellular vesicles (EVs)^114^; lipids can be recycled by microglia for export following phagocytosis of myelin debris.

Dysregulated lipogenesis leads to accumulation of toxic substrates that cause neuron degeneration, loss of myelin, and/or OL death. A balanced lipid ratio is crucial for the function and health of OLs. For instance, n-3 polyunsaturated fatty acids (n-3-FA) are pro-resolving lipid mediators. N-3-FA are produced from n-6-FA via Fat-1. Efficient conversion in Fat-1 controls low levels of n-6/n-3, which support OL generation during remyelination, although in part through regulation of microglial inflammation^115^. Moreover, extracellular lipids can also have toxic effects on OLs. Long-chain saturated free fatty acids (FFAs) produced by reactive astrocytes promote OL death in culture^116^. Oxidised forms of cholesterol (oxysterols) which accumulate in aging and AD promote OL death^117^. In addition, oxidized phosphatidyl cholines—markers of oxidative stress—are detected in MS lesions, mediate OL death in culture, and induce demyelination when injected in vivo^118^.

The importance of lipids in regulating OL responses is highlighted by the dysregulation of their synthesis or transport in neurological disorders characterized by OL injury and dysfunction. For instance, the cholesterol biosynthesis pathway is downregulated in MS and in astrocytes in the poorly remyelinating model EAE^119^, and single nuclei sequencing of chronic active MS lesions indicate altered lipid storage/response genes in microglia and astrocytes^47^. Decreased cholesterol levels observed in Smith-Lemli-Optiz Syndrome (SLOS) due to 7-dehydrocholesterol reductase (DHCR7) dysfunction are associated with abnormal developmental myelination^120,121^. Conversely, cholesterol accumulation can lead to hypomyelination and decompacted myelin as observed in some leukodystrophies. For instance, Pelizaeus-Merbacher disease is caused by a mutation in the myelin gene *PLP1*, which results in the accumulation of cholesterol and membrane raft components in lysosomal compartments due to mistrafficking of these myelin elements^122–125^. In addition, in Niemann-Pick disease type C, caused by mutations in regulators of cholesterol transport *NPC1* or *NPC2*, results in cholesterol accumulated in the lysosomes^126–128^. Cholesterol accumulation can also lead to demyelination, as seen in Cerebrotendinous Xanthomatosis, caused by mutations in the sterol 27-hydroxylase gene (*CYP27A1*)^129,130^. In addition, deficiencies in production of other lipids, such as sphingolipids, important for oligodendrocyte maturation, and fatty acids, lead to hypomyelination^131–135^.

### Growth factors and extracellular matrix regulation of OLs

Many extracellular factors have been implicated in regulating the responses of immature cells of the OL lineage, including IGF-1, LIF, CNTF, CSPGs, and laminins^136–139^. OLs are also influenced by neighbouring cells that shape the microenvironment by secreting growth factors and components of the extracellular matrix (ECM). For instance, platelet-derived growth factor (PDGF) can prevent apoptosis of newly formed OLs to support myelination and remyelination, while also supporting OPC proliferation and differentiation^140–145^. However, expression of its receptor PDGFRα needs to be tightly controlled, as it is normally downregulated with OL maturation and preventing this via overexpression drives myelin defects^146^. Fibroblast growth factor (FGF) signaling also regulates OLs, with FGFR2 ligation stimulating process outgrowth and regulating myelin thickness via ERK1/2 MAPK signaling^147,148^. Knockout of FGFR1 in mature OLs increases their numbers and promotes remyelination in EAE, while promoting myelination in vitro^149,150^. Of interest, FGF2 expression by astrocytes is increased in active MS lesions, and is known to downregulate myelin proteins, cause loss of mature OLs, and inhibit myelin formation^150–152^. ECM components are also important regulators of OL responses, and their dysregulation after injury contributes to remyelination failure^153,154^. Cell surface integrin receptors on OLs such as β1 integrin interact with ECM ligands on axons to amplify growth factor signaling, regulating the survival of pre-myelinating and myelinating OLs, increasing myelin membrane formation, influencing axonal selection for myelination in an diameter-dependent manner, and regulating myelin gene translation^155–160^. Astrocytes contribute to the ECM, as the main source of fibronectin after demyelination, which can inhibit OL differentiation and remyelination^161^; fibronectin is increased in the plasma of pre-symptomic and symptomatic people with MS, while low levels are observed in remyelinating MS lesions^161^. Astrocytes also produce hyaluronan during chronic demyelination, impairing remyelination by arresting OL differentiation via Toll-like receptor 2 ligation^162–164^; of note, hyaluronan is increased in several demyelinating diseases^162,163,165^. Moreover, ECM may influence OLs through regulation of tissue stiffness, which regulates OL differentiation^166^, and is increased in chronic demyelination yet decreased during remyelination^167^.

### Immune and glial cell interactions regulating OLs

OL responses are directly and indirectly influenced by cell-cell interactions with immune cells and other glia. For instance, differentiation of OL lineage cells generated from MS patient iPSCs occurs efficiently in vitro and in vivo^168^, yet is inhibited by exposure to peripheral blood mononuclear cell supernatants, with CD4+ T cell-derived interferon (IFN)-γ being a prime contributor^169^. IFNγ can also induce OPC death indirectly, via enhanced CD8+ T cell infiltration and subsequent antigen presentation by OPCs^99^. Primary human adult OLs undergo cell death in vitro following exposure to several T cell populations and natural killer cells, occurring in part through NKG2C/D receptor-ligand interactions^37,38^; OLs express the ligands in MS lesions thereby making them susceptible to immune mediated cytotoxicity^37,38^. Furthermore, direct contact of OLs with T helper (Th) 17 cells promotes glutamate release that alters OL cholesterol biosynthesis, thereby causing stress, reducing process formation, and increasing death^39^. In EAE, ultrastructural analysis demonstrated infiltrating monocytes appearing to pull myelin off the axon^170^; together with the known requirement of monocytes to initiate disease in EAE^171^, this suggests an important interaction between monocytes and myelin processes in inducing demyelination. Accordingly, macrophage depletion via small molecule inhibition of the pro-survival receptor CSF1R is sufficient to reduce OL loss and demyelination in the cuprizone model, and administration of the ligand CSF1 is sufficient to cause demyelination^172^. Microglia are in close communication with astrocytes to drive demyelination, as seen in EAE^13^, through secretion of pro-inflammatory cytokines, complement signaling^47^, and semaphorin and ephrin signaling^173^. Although direct mechanisms by which microglia interact with mature OLs to regulate their responses are not clear, a study in zebrafish showed that microglia engulf myelin sheaths to regulate sheath number in developmental myelination^174^. In addition, microglia in the presence of astrocytes induce OL death through TNFα after exposure to the pro-inflammatory stimuli lipopolysaccharide (LPS)^175^. Astrocytes may also contribute to demyelination indirectly via induction of BBB breakdown and expression of chemokines that recruit peripheral immune cells^176–180^. Human OLs are however resistant to toxic effects of conditioned media from astrocytes preconditioned by immune cell supernatants, whereas immature OL lineage cells are susceptible to impaired differentiation and/or death^181^. Conversely, immune cells can have protective effects on OLs. Astrocytes indirectly promote OL survival by inducing apoptosis in CD4+ T cells^182^, and peripheral immune cells in MS produce leukaemia inhibitor factor (LIF) which protects OLs from TNFα-induced death in vitro^183^. In addition, microglia and astrocytes support OL lineage responses during myelination and remyelination, in part via secretion of growth factors that can act on OL receptors^51,138,184,185^. Altogether these studies suggest that cell-cell interactions with OLs can have a major influence on OL and myelin pathology in neuroinflammatory diseases such as MS.

## Outlook

Therapies currently approved for myelin disorders such as MS are focused on dampening initial myelin damage via immune-modulation. Whereas these drugs can reduce the frequency of clinical relapses, they do not prevent MS progression and clinical decline, likely due to the continued impairment in remyelination and consequent axonal dysfunction and loss. This highlights the importance of complementing immune modulation with neuroprotective and pro-remyelination therapies^186,187^. Clinical trials are underway assessing drugs targeting neuronal survival and OPC differentiation into remyelinating OLs. We put forward that therapeutic targeting of mature OLs to reduce their injury and dysfunction is a complementary strategy that may support neural health. However, several factors need to be taken into consideration to achieve successful therapeutic targeting of OLs in neurological disease.

First, targeting of mature OLs without off-targets on the immature stages of the OL lineage may be important, given their differential susceptibilities to injury and responses to extrinsic factors. Although mature OLs were long considered not to contribute to remyelination, recent work has surprisingly demonstrated the contrary. Studies have demonstrably shown that OLs surviving demyelination can extend processes to remyelinate, as shown in cats, mouse, and zebrafish^188–190^. Consistent with these findings, ^14^C-labeling suggests that remyelinating MS lesions contain ‘old’ OLs^191^, implying that existing OLs mediated remyelination (although non-proliferating OPC-derived OLs may have also contributed). However, in an elegant study performed in zebrafish larvae enabling imaging of OL behavior in real time, Neely et al. revealed that OLs surviving demyelination remyelinate poorly compared to newly generated OLs, extending fewer processes and mistargeting myelin to neuronal cell bodies^190^; importantly, this mistargeted myelin profile was also found in MS lesions^190^. These studies suggest that therapeutic targeting of OL survival would need to also take into account the potential for ensuing poor quality remyelination. Revealing the mechanisms underpinning surviving OL-mediated remyelination, and how this can be improved, is key in harnessing the regenerative potential of these cells. This may be particularly important if drugs promoting OPC differentiation lead to eventual depletion of the progenitor pool, and considering that aged OPCs have decreased capacity to mature and remyelinate, and are less responsive to pro-remyelinating drugs^101^.

Second, one may need to consider targeting OL subpopulations. While specific functions of OL subpopulations remain to be elucidated, the shift in OL populations in disease may contribute to pathology and therefore may need to be considered for targeted therapy. For instance, an OL subpopulation with alteration in pathways related to inflammation and lipid metabolism has been found to emerge in several models of demyelination (toxin induced, autoimmune-mediated, and an amyloidosis model of Alzheimer’s disease), identified by high expression of *C4b* and *Serpina3n*^72,192–194^; accordingly, OLs expressing complement have been observed in numerous neurodegenerative diseases. Should this population be associated with increased vulnerability to injury or contribution to myelin pathology, targeting this subpopulation would be of interest. Moreover, the differential responses of the OL lineage in white vs gray matter, particularly in the context of remyelination, suggests the importance of considering distinct regional responses of OLs to therapeutics.

Third, sex differences in the OL lineage and myelination have been well described in rodents, although less so in humans^195,196^. Of note, MS is of lower prevalence but worse severity in males versus females. Although the OL subpopulation changes in MS could not be explained by sex differences^84^, the potential for differential responses to injury needs to be further investigated.

In summary, we put forward that targeting mature OLs is an untapped therapeutic avenue to support CNS health in the context of neurological disorders. This holds promise not only in the treatment of neurological disorders typically considered to be those of the white matter like MS, but also other neurodegenerative conditions where OL and myelin pathology have been described, such as Alzheimer’s disease, ALS, Huntington’s Disease, and Schizophrenia^27–29,197,198^. Furthering our understanding of the mechanisms driving sublethal and lethal injury of OLs is therefore a critical priority in expanding effective therapeutic options for a broad spectrum of neurological disorders across the lifespan.
